# Commonly missed nursing cares in the obstetrics and gynecologic wards of Tigray general hospitals; Northern Ethiopia

**DOI:** 10.1371/journal.pone.0225814

**Published:** 2019-12-23

**Authors:** Mebrahtom Haftu, Alem Girmay, Martha Gebremeskel, Gebrekiros Aregawi, Dawit Gebregziabher, Carmen Robles

**Affiliations:** 1 School of Nursing, College of Health Science, Aksum University, Aksum, Tigray, Ethiopia; 2 School of Nursing, College of Health Science, Mekelle University, Mekelle, Tigray, Ethiopia; 3 Midwifery Department, College of Health Science, Mekelle University, Mekelle, Tigray, Ethiopia; Hong Kong Polytechnic University, HONG KONG

## Abstract

**Background:**

Missed nursing care is considered an error of omission and is defined as any aspect of required patient care that is omitted (either in part or whole) or significantly delayed. Nursing care missed in the perinatal setting can cause negative outcomes and repercussions for the quality and safety of care. This has been reported in multiple settings and countries and is tied to negative maternal outcomes. Preventing missed nursing care requires in-depth research considering the clinical setting.

**Objective:**

The main aim of the study was to assess commonly missed nursing care elements, reasons, and factors for the omission in the obstetric and gynecologic units of general hospitals in Tigray 2017/18.

**Methods and materials:**

A cross-sectional study was conducted in eight randomly selected general hospitals in Tigray, Ethiopia. A total of 422 nurses and midwives were selected through simple random sampling using the staff list as a sampling frame. To identify the commonly missed nursing care and related factors, the MISSCARE survey tool was used. Descriptive, bivariate, and multivariate logistic regression analysis was performed to assess potential risk factors of nursing cares omission.

**Result:**

The study results showed that 299 (74.6%) participants commonly missed at least one nursing care in the perinatal setting. Labor resources 386(96.3%), teamwork 365(91%), material resources 361 (90%) and communication 342 (85.3%) were the reasons identified for commonly missing care. In the multivariate analyses, sex (p-value <0.001), educational level (p-value 0.034), working shift (p-value <0.001) and having an intention to leave the institution (p-value <0.001) showed a significant association with commonly missing care.

**Conclusion:**

The proportion of commonly missed nursing care was high. After adjusting for demographic variables, labor resources, material resources, and communication were reasons for commonly missed nursing care. Increasing male professional proportion, investing in nurses/midwives training, and harmonizing nursing service administration through appropriate working shift arrangement and timely assessment of professionals’ stability and satisfaction could minimize frequent omission of nursing care.

## Introduction

Conceptually, missed nursing care is considered an error of omission and is defined as any aspect of required patient care that is omitted (either in part or whole) or significantly delayed [1]. Over the last two decades, many studies have been published supporting the hypothesis that the quality and quantity of nursing care contribute directly and indirectly to outcomes such as morbidity and mortality, failure to rescue, hospital length of stay (LOS), hospital readmission, and patient satisfaction in hospitalized patients [2–4].Missed nursing care was only recently recognized as a widespread concern within the nursing and midwifery disciplines. Ensuring quality nursing care and patient safety is a major challenge facing nurses and midwifery leaders today and it is not commonly recognized that required nursing care is often left undone [2, 5, 6]. However, evidence suggests that 9 out of 10 nurses miss some essential care activities each shift [1]. Estimates of the prevalence of missed care are high (55–98%), depending on the instrument used, among nursing and midwifery staff in acute care hospitals of multiple settings and countries [3, 5]. According to the literature, this problem is high even in the developed world. In Sweden, 74% of hospital nurses miss nursing care [7]. In the United States of America (USA) important nursing care omission ranges from 10% to27% in all settings [8].

Studies conducted on nursing care omission mainly cover the emergency, critical care unit, medical, and surgical units. The most frequent omission was documented in nursing care elements like ambulation, mouth care, medication timing, and patient turning [4, 7, 9]. Labor resources are the main reason for commonly missed nursing care. Hospital system factors such as working shift [7, 10], wages and the career structure of institutions also affect the professional’s performance as it determines stability, i.e reduce the likelyhood of an intention to leave [8, 11, 12]. Beyond this, nursing care omission is determined by professional’s characteristics like gender stereotype [13–16], level of education [17], experience, knowledge, and attitude.

Ethiopian nurses and midwives are exposed to many work-related challenges that may influence the quality of nursing care. Challenges include staff shortages, working immediately after graduation with no experience, working overtime or having two or three different jobs. In addition poor collaboration with other healthcare professionals is higher compared to other African countries [12, 18].

Ethiopian hospital’s overall implementation of nursing/midwifery care standard practice is still just 48.2%. In the perinatal setting, the implementation is less than 20%. Poor implementation of the nursing care plan and absence of clear job distribution are problems in perinatal settings[19, 20]. Though midwives are mainly responsible for care given in labor and delivery units, and nurses oversee care given in gynecology wards, dysfunctional job distribution is has caused conflicting roles among nurses/midwives. According to one systematic review and meta-analyses, the estimated pooled level of patient satisfaction with nursing care in Ethiopia is 55% [21].

In the developing world, literature that documents nursing care omission is scant. In the perinatal setting, though it is not widely assessed, it would have a positive effect on the health-seeking behavior of pregnant mothers and their utilization of antenatal and postnatal care [2, 6]. The high maternal mortality ratio in Ethiopia and other developing countries reflects these problems[2, 22, 23]. So identifying care missed and factors related to these omissions in developing countries like Ethiopia will contribute to the improvement of nursing care service in the perinatal setting.

## Materials and methods

An Institution-based cross-sectional study was conducted between October and December 2017 among general hospitals of Tigray northern Ethiopia. There were 16 general hospitals in the study area, and during the study period, there were 3067 nurses and 792 midwives assigned in the hospitals. All nurses and midwives who work in the obstetrics and gynecology unit of Tigray general hospital served as source populations.

### Sample size determination and sampling

The sample size was calculated using a single population proportion formula by considering the proportion of missed nursing care in the perinatal setting of general hospitals as 50% with a 5% margin of error and a 95% confidence level.

n=(Zα2)2P(1−P)d2=(1.96)20.5(1−0.5)0.052=384

Considering a 10% contingency for non-response, the survey was conducted on 422 professionals. However, only 401 (95%) participants completed questionnaires. Participants were selected from eight randomly selected general hospitals out of the sixteen general hospitals in Tigray and the actual participants were randomly selected through computer-generated numbers using the staff list as the sampling frame.

### Data collection procedures

Data was collected using the MISSCARE survey tool which was adopted from Kalisch BJ [9]. It is an instrument that measures missed nursing care elements and the reasons for the nursing care omission. The questionnaires consisted of 53 questions divided into three parts. Part one contains socio-demographic questions. Part two contains 26 questions that assess the frequency of nursing care element omission. The response range consisted of a Likert scale with the following answers: 0 does not apply, 1 never, 2 rarely, 3 sometimes, 4 frequently and 5 always missed. Part three assessed reasons for the omission of nursing care. Eighteen questions were asked in four categories: labor resources (6 questions), material resources (3 questions), teamwork (5 questions), and communications (4 questions). The response range consists of 1–4; one if no reason, two minor reasons, three for a moderate reason, and four significant reasons. The tool was developed through an analysis of interview data, a review of literature and interviews with key informants, and then tested on two occasions to determine and construct validity and reliability of the instrument [1]. A modification was made by maternal health experts to assess the relevance of nursing care elements to the perinatal units.

### Study variables

Commonly missed nursing care was the dependent variable. In this study, commonly missed care is defined as any aspect of required patient care that is often or always omitted (either in part or in whole) or significantly delayed (if the care was conducted after it becomes no longer necessary). Socio-demographic characteristics of professionals, staff outcomes (satisfaction, turnover, intent to leave), unit characteristics e.g. type of nurse and midwives staffing, and teamwork (communication within and other discipline) were the independent variables.

### Data analysis procedures

The collected data were double entered into a computer using Epi-info version 7 and exported into SPSS version-22 for analysis. For our analyses, response alternatives on missed nursing care were transformed into a dichotomous scale, in which the alternatives 1, 2 and 3 were considered as care provided, and alternatives 4 and 5 were considered as care commonly missed [9, 24]. Descriptive statistics summarized factors relating to care lost using frequencies, percentages as well as mean and SD. Bivariate logistic regression was used to determine the association between independent and dependent variables and those variables with a p-value < 0.2 were included in a multivariate logistic regression in order to control the possible confounders. Before inclusion of predictors to the final logistic regression model, the multi-collinearity effect was checked using VIF/Tolerance test. The Hosmer-Lemeshow goodness-of-fit statistic was used to check if the data fit the logistic model. Adjusted Odds ratio with 95% confidence interval for those variables with p-value < 0.05 was calculated to show the level of association and statistical significance.

### Ethical considerations

Ethical clearance was received from Aksum University Collage of Health Science Health Research Ethics Review Committee (IRB: 041/2017) and full written informed consent was obtained from participants. Privacy and strict confidentiality were maintained during the data collection process. No personal details were recorded or produced on any documentation related to the study.

## Results

The response rate was 95%. The analysis was performed on the 401 completed questionnaires. Most participants were females. The mean age of respondents was 29 ± 5.06 years, with ages ranging from 23 to 45 years. Most of the participants were BSc degree holders. Results also showed that over two-fifth had from one up to five years of experience, and four out of five participants mostly worked a day shift. Only one-fifth of the professionals were satisfied with the payment they were receiving, and the majority had an intention to leave the institution (Table 1).

**Table 1:** Socio demographic and personal characteristics of participants (N = 401).

Variable	Frequency N	Percent (%)
**Sex**		
Male	146	36.4
Female	255	63.6
**Educational status**		
Diploma	30	7.5
Degree	342	85.3
Masters	29	7.2
**Profession**		
Nurse	167	42.6
Midwives	234	58.4
**Job experience**		
Less than one year	7	1.7
One up to five years	246	61.3
More than five years	148	36.9
**Absent for >2 days within 3 months**		
Yes	96	23.9
No	305	76.1
**Shift mostly worked**		
Day	332	82.8
Night	69	17.2
**Institution Graduated from**		
Private institution	126	31.4
Governmental	275	68.6
**Satisfied with the payment**		
Yes	79	19.7
No	322	80.3
**Intent to leave institution**		
Yes	287	71.6
No	114	28.4

### Commonly missed nursing cares

Distribution of responses on how frequently each element of care was commonly missed by the professionals showed that 299 (74.6%) reported that they commonly missed at least one nursing care. Several nursing care activities were reported as frequently or often missed; among them, complete head to toe physical examination was the most frequently missed element reported by 148 (36.9%) of the participants, followed by ongoing and timely monitoring of patient status 137 (34.2%), continuous intake and output measurement 133 (33.2%), giving an immediate response to patients rapidly changing condition or deterioration 123 (30.7%) and giving a reassurance to the mother 122 (30.4%) On the other hand, the least frequently reported commonly missed nursing care activities were; reviewing laboratory results, giving reassurance to the family, discussing about the mothers expectation, giving an explanation mothers about the test and diagnosis, which were reported by 70 (17.5%), 73 (18.2%), 77 (19.2%) and 80 (20%) of the participants respectively (Table 2).

**Table 2:** Results on frequency of nursing care elements omission (n = 401).

Nursing Care activity		
Elements	Commonly missed N (%)	Performed N (%)
Physical examination (head-to-toe)	148(36.9)	253(63.1)
Ongoing and timely monitoring of patient status	137(34.2)	264(65.8)
Intake and output measure	133(33.2)	268(66.8)
Response to rapidly changing condition or deterioration	123(30.7)	278(69.3)
Reassuring the mother	122(30.4)	279(69.9)
Documentation	120(29.9)	281(70.1)
Timely Nurse/Midwives to patient communication	118(29.4)	283(70.6)
Complete review of history	117(29.2)	284(70.8)
General comfort care based on patient need	115(28.7)	286(71.3)
Repositioning when patients needed	112(27.9)	289(72.1)
Timely cervical examinations	111(27.7)	290(72.3)
Labor support	109(27.2)	292(72.8)
Continuous history taking (clerking)	108(26.9)	293(73.1)
Medications given immediately postpartum	106(26.4)	295(73.6)
Answering questions raised by patient	104(25.9)	294(74.1)
Initial assessments (physical, social, emotional, psychological)	100(24.9)	301(75.1)
Handoffs at every shift	97(24.2)	304(75.8)
Bedside presence “Being with” the woman	96(23.9)	305(76.1)
Developing a plan of care	94(23.4)	307(76.6)
Medication administration for labor	93(23.2)	308(76.8)
Monitoring FHR	89(22.2)	312(77.8)
Pain management	82(20.4)	319(79.6)
Teaching about procedures, tests diagnostic studies	80(20)	321(80)
Discussing about patients expectation	77(19.2)	324(80.2)
Reassuring the family	73(18.2)	328(81.8)
Review of lab results obtained during labor and birth	70(17.5)	331(82.5)

### Reasons for commonly missing a nursing care

The current study shows that labor resources was the primary reason for commonly omitting or delaying a nursing care 386 (96.3%), followed by teamwork 365 (91%), material resources 361 (90%), and communication 342(85.3%). In the labor resources subscale, the most commonly reported items were lack of experience or previous exposure (88.1%) and unexpected rise in patient volume (86.8%). Among the material resources, shortage of supplies and equipment’s when needed (89.5%), and from the teamwork resources category, lack of backup support from team members when needed (84.5%) was the prominently reported reasons (Table 3).

**Table 3:** Results on reason for commonly missing a nursing care in the perinatal setting (N = 401).

Reason category	Reason sub-scale	Reasoning scale			
		Not a reason	Minor reason	Moderate reason	Significant reason
	Inadequate number of staff	91(22.7)	76(19)	153(38.2)	81(20.2)
	Urgency of patient situations/ patient's condition worsening	52(13)	153(38.2)	115(28.7)	81(20.2)
**Labor Resources**	Unexpected rise in patient volume	53(13.2)	149(37.2)	94(23.4)	105(26.2)
	Inadequate number of assistive personnel	74(18.5)	110(27.4)	139(34.7)	78(19.5)
	Heavy admission and discharge activity	103(25.7)	84(20.9)	131(32.7)	83(20.7)
	Lack of experience or previous exposure	48 (11.9)	128(31.9)	110(27.4)	115(28.7)
**Material Resources**	Medications were not available when needed	55(13.7)	150(37.4)	81(20.2)	115(28.7)
	Supplies/equipment not available when needed	42(10.5)	97(24.2)	136(33.9)	126(31.4)
	Supplies/equipment not functioning properly when needed	49(12.2)	95(23.7)	15237.9	105(26.2)
**Team work**	Unbalanced patient assignments	86(21.4)	128(31.9)	117(29.2)	70(17.5)
	Inadequate hand-off from previous shift or sending unit	71(17.7)	178(44.4)	113(28.2)	39(9.7)
	Other departments/unites did not provide the care needed	71(17.7)	155(38.7)	108(26.9)	67(16.7)
	Lack of back up support from team member	66(16.5)	149(37.2)	138(34.4)	48(12)
	Caregiver go off unit or unavailable	98(24.4)	137(34.2)	119(29.7)	47(11.7)
**Communication**	Tension or communication breakdowns with other departments	53(13.2)	143(35.7)	148(36.9)	57(14.2)
	Tension or communication breakdowns within the team	120(29.9)	88(21.9	107(26.7)	86(21.4)
	Tension or communication breakdowns with the medical staff	89(22.2)	114(28.4)	114(28.4)	84(20.9)
	Nursing/Midwifery assistant did not communicate if that care was not done	74(18.5)	104(25.9)	103(25.7)	120(29.9)

### Factors associated with commonly missed nursing cares

Logistic regression analysis was done to identify the commonly missed nursing care elements and variables that can independently affect nurses’ and midwives’ behavior so as to commonly miss care in the perinatal setting. All variables with a p-value < 0.2 in the bivariate analyses were used in multivariate logistic regression in further analyses to control for confounders. Participant’s sex, educational level, working shift, and having an intention to leave the institution were factors that showed a significant association in this multivariate analysis.

In the multivariate analysis, four variables had significant effects. Male professionals had lower odds to miss care commonly compared to female professionals. (AOR: 0.20, 95% CI: (0.10, 0.38)). The second variable was educational level. Those who had a bachelor's degree were sixteen times higher (p-value, 0.034) and those who had diploma were five times higher (p-value, < 0.001) to miss nursing care commonly than those who had a master’s degree. The third variable that showed an association was working shift. Professionals that mostly work in the night shift were about six times more likely to commonly miss nursing care as compared to those who work in the day shift. The fourth variable was having an intention to leave the institution, which increased the odds of missing nursing care commonly by 2.33 times compared to being stable (Table 4).

**Table 4:** Bivariate and multivariate binary logistic results on factors that affect nursing care omission commonly.

Variable	Commonly Miss Care		COR(95%CI)	AOR(95% CI)	P- Value
	Yes N (%)	NO N (%)			
**Sex**					
Male	98(32.8)	48(47.1)	0.55(0.35,0.87)	0.20(0.10,0.38)	**<0.001**
Female	201(67.2)	54(52.9)	1	1	
**Educational status**					
Diploma	16(5.4)	14(13.7)	3.6(1.18,10.9)	5.06(1.13,22.7)	**0.034**
Degree	276(92.3)	66(64.7)	13.1(5.4,32.0)	16.7(5.35,52.2)	**<0.001**
Masters	7(2.3)	22(21.6)	1	1	
**Job experience**					
< one year	22(7.4)	16(15.7)	0.54(0.25,1.13)	0.86(0.30,2.43)	0.77
1–5 years	178(59.5)	47(46.1)	1.49(0.91,2.43)	1.86(0.89,3.86)	0.09
5 years & more	99(33.1)	39(38.2)	1	1	
**Two or > Absent within 3 months**					
No	222(74.2)	83(81.4)	0.66(0.38,1.16)	0.45(0.21,1.94)	0.34
Yes	77(25.8)	19(18.6)	1	1	
**Shift type that you mostly worked**					
Night	256(85.6)	76(74.5)	2.04(1.17,3.53)	6.05(2.56,14.3)	**<0.001**
Day	43(14.4)	26(25.5)	1	1	
**Institution Graduated from**					
Private	104(34.8)	22(21.6)	1.94(1.14,3.29)	3.0(0.43,6.33)	0.54
Governmental	195(65.2)	80(78.4)	1	1	
**Marital status**					
Single	159(53.2)	46(45.1)	1.38(0.88,2.17)	1.38(0.67,2.82)	0.37
Married	140(46.8)	56(54.9)	1	1	
**Experienced professional are Leaving**					
Yes	252(84.3)	98(96.1)	0.21(0.07,0.62)	0.13(0.04,1.41)	0.110
No	47(15.7)	4(3.9)	1	1	
**Satisfied with the payment**					
No	238(79.6)	84(82.4)	0.83(0.46,1.49)	0.96(0.43,2.12)	0.92
Yes	61(20.4)	18(17.6)	1	1	
**Intent to leave institution**					
Yes	231(77.3)	56(54.9)	2.79(1.73,4.48)	4.6(2.33,9.09)	**<0.001**
No	68(22.7)	46(45.1)	1	1	

## Discussion

In any setting, missing essential aspects of nursing care is common especially when the environment is busy. Regardless of the workload, a tremendous element of nursing care is left undone in different settings and for different reasons [1, 23]. In this study 74.6% of the participants commonly miss at least one important nursing care in the Obstetrics and Gynecology ward per shift. These findings are comparable to a study from Sweden which resulted in 74% [7] and the first quantitative studies conducted using the MISSCARE Surveys 70% [1, 4]. However, the magnitude of nursing care omission was higher compared to the study conducted in New Jersey USA (10–27%)[8]. This difference might be due to the study setting and sample size difference. Moreover, the increased magnitude of nursing care omission could be related to the fact that nurses/midwives do not prioritize these interventions, give low emphasis to the importance of the nursing care element, or they consider these tasks manageable by other colleagues or relatives of the patient [25].

The five most frequently missed elements in the perinatal setting are: complete head-to-toe physical examination; ongoing and timely monitoring of patient status; continuous intake and output measurements; immediate response to a patient’s rapidly changing condition or deterioration; and providing reassurance to the mother. These findings revealed that the maternal condition was neglected and the caregiver’s attention might be with the newborn; this is evidenced by the increased maternal mortality ratio in Ethiopia and other developing countries [22].

Nursing care activities such as reviewing laboratory results, providing reassurance to family, discussing the mother's expectations, giving explanations, and teaching mothers about tests and diagnoses were considered to be the least frequently reported elements. This result is clear especially in the clinical perspective because cares are audited and cross-checked by unit heads and other professionals such as physicians. This could be the reason for the less frequent omission [8].

In the reason’s assessment for commonly missing nursing care: Labor resource is the most quoted reason. Within the labor resource subscale, unexpected rise in a patient volume cited as the top cause for missed cares[1, 3, 26]. Study results reveal labor resources as one of the factors, with increased patient volume being the next; but inadequate work experience and exposure was the prominent reason. This shows that the staff lacks competency regarding standard nursing care implementation in the clinical setting. This problem might be related to professional dissatisfaction, unsatisfactory rotation schedule and low emphasis given for professional development in the study area[12].

Findings from this study reveal significant correlates of nursing care omission. Among those professionals, gender is one. Nursing and midwifery remain a female-dominated profession around the world and gender stereotyping is still a major issue in developing countries. Few studies report the situation of novice male nurses even in their first year of service[13, 16]. Supporting this evidence in this study, male professionals were less likely to commonly miss care compared to female professionals (AOR: 0.20, 95% CI: (0.10, 0.38). The most cited reason was the male concern about their career options and promotion. Most male nurses/midwives are unhappy if they deliver less advanced care. Thus, immediately after deployment to the professional practice, male nurses/midwives typically concentrate on delivering a good standard of service and earning a promotion [13, 16, 27]. This possibly helps them overlook essential cares less frequently than their female counterparts [13, 16].

Regarding educational level, in this study diploma, and bachelor professionals as compared to the master’s degree holders reported higher rates of missed care. This result is in line with the previous study findings, revealing that lower-level nurses/midwives on duty increase the likelihood of many aspects of care being delayed or left undone [17]. This could be because of the fact that level of education determines the exposure, experience, knowledge, and attitude of these professionals. But in this study individual that holds a bachelor’s miss an element of care more commonly than the diploma degree holders; This could be because of the existing problem in the job description, which leaves bachelor holders inexperienced and less supervised.

Another factor that showed an association was the working shift. Professionals that work mostly during the night shift were six times more likely to miss nursing care as compared to those who worked during the day shift. This result is in line with earlier literature [7, 10]. The science that describes the effect of diurnal variation is the most commonly cited reason for poor performance in the night shift. Working at night can frequently induce sleep disorder because of short circadian rhythms of cortisol secretions which could influence mental and physical health; leading them to miss important care.

Stability in the working environment is another factor. Those who had intentions to leave their institution were 2.33 times more likely to miss nursing care than those who do not. This result is also supported by the literature [11]. Those who intended to leave their institution are possibly investing their time in searching for a vacancy or doing overtime work. This might deteriorate their concern about their care performance and their absenteeism which might then lead them to miss nursing care.

The study findings of this study are pertinent to the field as it will allow nursing and midwifery managers to make decisions that will strengthen nursing care. Still, besides its significance, the study had drawbacks. We conducted the study through self-administered questionnaires; therefore, the study was assessed by self-report. Because of this, under-reporting of the nursing omission might affect the result. Study participants were nurses and midwives hence lacking patients’ opinions that would provide greater clarity in the phenomenon of missed care. As the study was cross-sectional, the result does not show the impact of missed perinatal care on the mother and infant.

## Conclusion

Most of the nurses and midwives working in the Obstetrics and Gynecology wards were commonly missing nursing care elements. The magnitude of nursing care omission was high in the study data. Labor resources were one of the reasons for missing nursing care. Regional Health Bureau and Hospital Administration, which requires strengthening the support and supervision on implementing the nursing care plan to minimize the omission. Professionals of the female gender, of a lower educational status, working in night shift, and having an intention to leave the institution were also independent predictors. Consequently, increasing the proportion of male professionals, investing in nurses’ and midwives’ education, and harmonizing nursing service administration could reduce the frequency of omissions in nursing care. Nurse and Midwife managers also need to assess professionals’ stability and satisfaction to enhance nurses’ and midwives’ working capacities. To assess the impact of the commonly missed nursing care elements, researchers need to focus on a further longitudinal follow up study.

## Supporting information

S1 Dataset (SAV)

S1 Fig (TIFF)

## References

[1] Ball JE, Griffiths P, Rafferty AM, Lindqvist R, Murrells T, Tishelman C A cross-sectional study of ‘care left undone’ on nursing shifts in hospitals. Journal of Advanced Nursing 2016: 72: 2086–2097. 10.1111/jan.12976 27095463

[2] Kalisch BJ, Landstrom G, Hinshaw AS. Missed nursing care: A concept analysis. J Adv Nurs.2009;65(7):1509–17. 10.1111/j.1365-2648.2009.05027.x 19456994

[3] Kalisch BJ, Tschannen D, Lee KH. Do staffing levels predict missed nursing care? Int J Qual Health Care.2011;23(3):302–308 10.1093/intqhc/mzr009 21486856

[4] Moreno-Monsiváis MG, Moreno-Rodríguez C, Interial Guzmán MGMissed Nursing Care in Hospitalized Patients. Aquichan.2015;15(3):318–28. 10.5294/aqui.2015.15.3.2

[5] Jones Terry L., Hamilton Patti, Murry Nicole. Unfinished nursing care, missed care, and implicitly rationed care: State of the science review International Journal of Nursing Studies 2015 52:1121–1137 10.1016/j.ijnurstu.2015.02.012 25794946

[6] Aiken LH, Clarke SP, Sloane DM, Sochalski J, Silber JH. Hospital nurse staffing and patient mortality, nurse burnout, and job dissatisfaction. JAMA.2002;288 (16):1987–1993. 10.1001/jama.288.16.1987 12387650

[7] Ball JE, Murrells T, Rafferty AM, Morrow E, Griffiths P. “Care left undone” during nursing shifts: associations with workload and perceived quality of care. BMJ Qual Saf.2013 10.1136/bmjqs-2012-001767 23898215 PMC3913111

[8] Hessels AJ, Flynn L, Cimiotti JP, Cadmus E, Gershon RR. The Impact of the Nursing Practice Environment on Missed Nursing Care, J Clin Nurs Stud, 20153(4): 60–65. 10.5430/cns.v3n4p60 27547768 PMC4988676

[9] Kalisch BJ, Williams RA. Development and psychometric testing of a tool to measure missed nursing care. J Nurs Admin.2009;39(5):211–9. 10.1097/NNA.0b013e3181a23cf5 19423986

[10] Kalisch B., Tschannen D., Lee H., & Friese C. R. Hospital variation in missed nursing care. American Journal of Medical Quality,2011 26, 291–299. 10.1177/1062860610395929 21642601 PMC3137698

[11] Tschannen D, Kalisch BJ, Lee KH. Missed nursing care: the impact on intention to leave and turnover, Can J Nurs Res 201042(4):22–39. 21319636

[12] Kebede M, Endris Y, Zegeye DT. Nursing care documentation practice: The unfinished task of nursing care in the University of Gondar Hospital 2016 10.1080/17538157.2016.1252766 27918228

[13] WenZhang Yi-LanLiu. Demonstration of caring by males in clinical practice: A literature review, International Journal of Nursing Sciences 2016: 3(3), 323–327

[14] Evans J.A. Cautious caregivers: gender stereotypes and the sexualization of men nurses' touch J Adv Nurs, 2002 40 (4)441–448 10.1046/j.1365-2648.2002.02392.x 12421403

[15] Patterson B.J, Morin K.H. Perceptions of the maternal-child clinical rotation: the male student nurse experience J Nur Educ, 2002 41 (6)266–272 10.3928/0148-4834-20020601-07 12096775

[16] Paterson B.L. Tschikota S Crawford M. Saydak M., Venkatesh P, Aronowitz T. Learning to care: gender issues for male nursing students Can J Nurs Res, 1996 28(1):25–39. 8717794

[17] Recio-Saucedo A, Dll’Ora C, Maruotti A, Ball J, Briggs J, Meredith P, et al What impact does nursing care left undone have on patient outcomes? Review of the literature, JCN, 2018 27 2248–2259. 10.1111/jocn.14058 28859254 PMC6001747

[18] Chaka B. Adult patient satisfaction with nursing care in. Addis Ababa hospitals: 2005 16 (4): pp.337–44.

[19] Haftom «Improving the implementation of nursing care process»: Mekelle Hospital, Ethiopia. 2013

[20] Melesse B: Patient waiting time and its determinants in the general outpatient department»: Debremarkos and Felegehiywot Referral Hospitals; Amhara regional state, North west, Ethiopia.2015.

[21] Mulugeta H, Wagnew F, Dessie G, Biresaw H, Habtewold TD. Patient satisfaction with nursing care in Ethiopia: a systematic review and meta-analysis. BMC Nursing.2019;18 10.1186/s12912-0348-9 PMC6615179 31320836

[22] FMOH. Ethiopian Health and health-related indicators Version 1. Addis Ababa Ethiopia: Federal Ministry of health; 2015September 2016.

[23] Kelly L. Vincent D. The dimensions of nursing surveillance: a concept analysis. J Adv Nurs.2011;67(3):652–61. 10.1111/j.1365-2648.2010.05525.x 21129007 PMC3242365

[24] Watson R. Lea A. The caring dimensions inventory (CDI): content validity, reliability and scaling J Adv Nurs, 1997,25(1): 87–94. 10.1046/j.1365-2648.1997.1997025087.x 9004015

[25] Kalisch BJ, Lee KH. Missed nursing care: Magnet versus non-Magnet hospitals. J Nurs Outlook.2012;60(5):32–9. 10.1016/j.outlook.2012.04.006 22824471

[26] Carter D. Nursing care left undone in European hospitals. Am J Nurs.2014;114(2)17 10.1097/01.NAJ.0000443762.89516.81 24481353

[27] Cheng M-L, Tseng Ying-Hua, Hodges E, Chou F-H. Lived Experiences of Novice Male Nurses in Taiwan. Journal of Transcultural Nursing.2018;29(1):46–53. 10.1177/1043659616676318 27815552

