# Supplementary figures and images for: Commonly missed nursing cares in the obstetrics and gynecologic wards of Tigray general hospitals; Northern Ethiopia
Source: PLoS One. 2019 Dec 23;14(12):e0225814. doi: 10.1371/journal.pone.0225814 (PMC6927650; doi:10.1371/journal.pone.0225814)

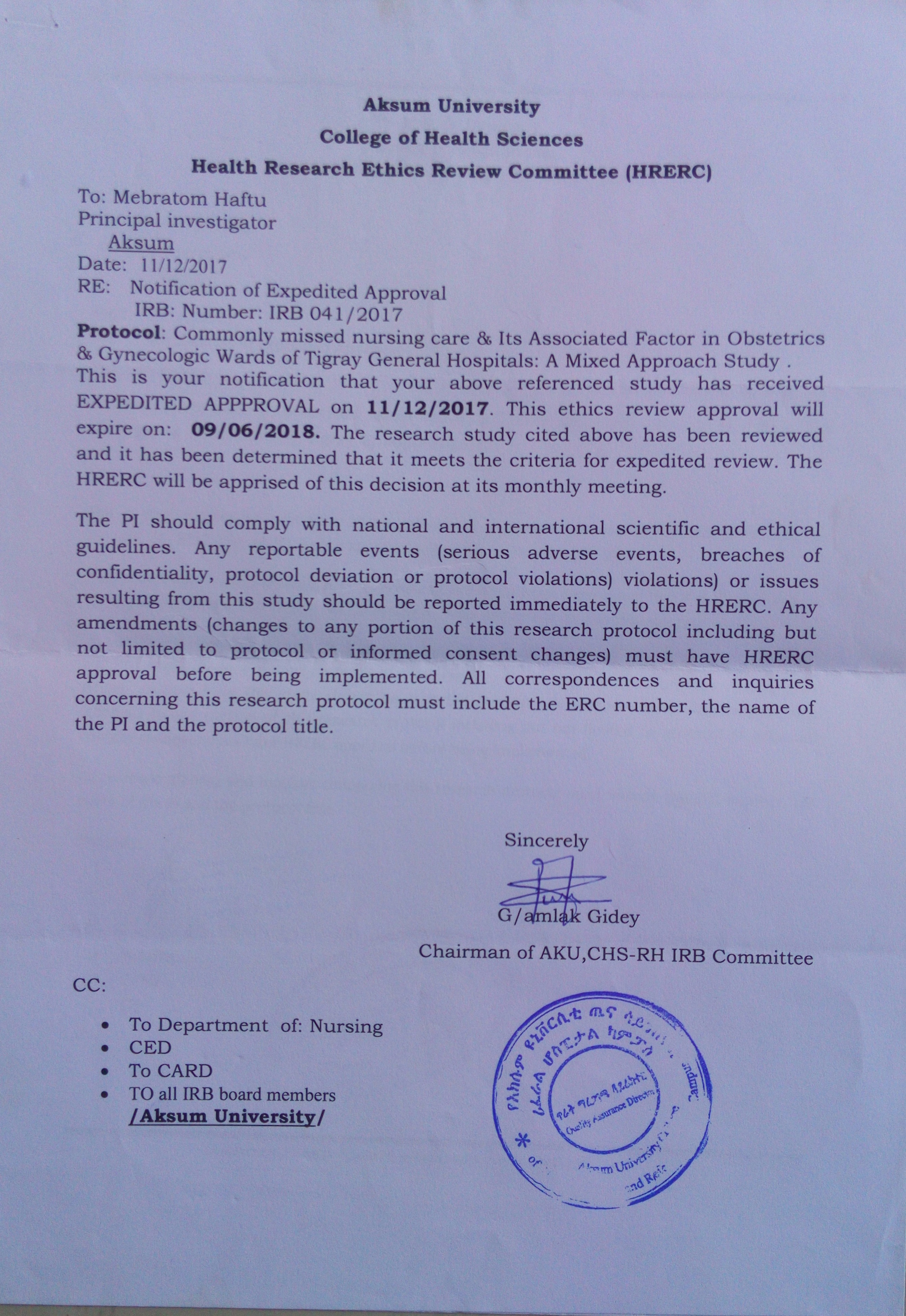

Supplement: S1 Fig — (TIFF) [file pone.0225814.s002.tiff]
